# Suppression of NF-κB activity by mutant IκBα: A molecular target for radiosensitization of adenoid cystic carcinoma

**DOI:** 10.3892/ol.2013.1153

**Published:** 2013-01-24

**Authors:** ZHE LIU, SHENGYUN HUANG, SHIZHOU ZHANG, JIAWEN SI, QIANG WANG, QIANGXIU WANG, WENLI MU, JUNQING HAN, DONGSHENG ZHANG

**Affiliations:** 1 Departments of Oral and Maxillofacial Surgery, Provincial Hospital Affiliated to Shandong University, Jinan 250021, P.R. China; 2 Pathology, Provincial Hospital Affiliated to Shandong University, Jinan 250021, P.R. China; 3 Medical Research Center, Provincial Hospital Affiliated to Shandong University, Jinan 250021, P.R. China; 4 Cancer Center, Provincial Hospital Affiliated to Shandong University, Jinan 250021, P.R. China

**Keywords:** nuclear factor κB, radiosensitization, apoptosis, transfection, ACC-M cells, pathway analysis

## Abstract

The constitutive activation of the nuclear factor κB (NF-κB) signaling pathway is involved in oncogenesis, invasive growth, metastasis and induced resistance to radiation and chemotherapy. Selective inhibition of the NF-κB signaling pathway, either by a mutant inhibitor or pharmacological agents, improves the therapeutic efficiency of irradiation. In the present study, the changes in NF-κB expression and the rate of apoptosis were investigated following irradiation of cells of an adenoid cystic carcinoma cell line (ACC-M) in which NF-κB expression had been inhibited by transient transfection with a mutant IκBα plasmid. ACC-M cells were transiently transfected with the mutant IκBα plasmid using Lipofectamine and the expression of this mutant IκBα gene was verified. The presence of the mutant IκBα gene alone did not result in a reduction in cell proliferation. Furthermore, a significant inhibition of translocation and synthesis of NF-κB protein in the transfected cells was observed after irradiation. NF-κB protein was activated by different doses of irradiation in a dose- and time-dependent manner with concordant changes in the radiosensitivity of ACC-M cells. We conclude that the mutant IκBα gene selectively inhibited the NF-κB pathway, which may be a promising method to improve the radiosensitivity of adenoid cystic carcinomas.

## Introduction

Adenoid cystic carcinoma (ACC) is one of the most common subtypes of malignant tumors occurring in the salivary glands. It is characterized by a high rate of recurrence, a strong tendency of perineural invasion and an early development of hematogenous metastasis. ACC patients possessing prognostic factors, including lymph node positivity, a solid histological subtype or perineural invasion with involvement of a named nerve, suffer poor prognostic survival propsects (1). The rates of perineural invasion and distant metastases after 10 years, regardless of aggressive surgery, have been demonstrated to be 50 and 39%, respectively (2). Postoperative radiotherapy is thus recommended for its additive improvement in the control of local and regional recurrence compared with surgery alone (1,3). However, considering the relatively low radiosensitivity and the aggressive growth pattern of ACC, methods to improve the therapeutic efficiency have potential for further investigation.

Nuclear factor κB (NF-κB) was first discovered in 1986 as a eukaryotic transcription factor that bound to the κ immunoglobulin light chain enhancer in the nuclei of the B lymphoid cell lineage, and is presently considered to exist in the majority of cell types (4,5). Homo- and heterodimeric complexes of its five members, p50, p52, p65/RelA, c-Rel and Rel-B, are sequestered inactive in the cytoplasm preformed and bound to the inhibitory subunits of the IκB family (IκBα, IκBβ, IκBε, Bcl-3, p100 and p105) (6). In the canonical NF-κB activation pathway, the activation of NF-κB depends on stimuli including bacterial and viral products, proinflammatory cytokines and ionizing radiation, all of which intrigue phosphorylation of IκBα by IκB kinase (IκK)-induced ubiquitination and subsequent proteolysis by the 26 S proteasome. The phosphorylation of IκBα at specific serine (Ser) residues liberates the captive cytoplasmic NF-κB for translocation to the nucleus and initiation of target genetic transcription (7). The active NF-κB transcription factor regulates the expression of >150 downstream genes. The majority of proteins encoded by NF-κB target genes participate in a wide variety of physiological processes, including embryonic development, lymphoid differentiation, immune and inflammatory responses and apoptotic resistance to radio- and chemotherapy, as well as being involved in oncogenesis and tumor proliferation (8).

The abarrent activation of NF-κB is characteristic of numerous solid tumor types, including breast epithelial tumors, pancreatic adenocarcinoma and bladder, prostate and non-small cell lung cancer (9). High levels of NF-κB activity contribute to a negative prognosis by playing a key role in the induction of anti-apoptosis, the acceleration of cell cycle progression, increased resistance to radiation and chemotherapy, as well as in elevated aggressive growth and metastatic frequency in malignant cancer (10). It is widely accepted that constitutively activating NF-κB exerts a negative impact on the radiosensitivity of different cancer cell lines. Sandur *et al* demonstrated that suppression of the NF-κB pathway via curcumin-induced inhibtion sensitized colorectal cancer cells to radiation by downregulating the phosphorylation and degradation of IκBα, inhibition of IκK activity and inhibition of Akt phosphorylation (10). By transfecting a gene encoding mutated IκBα that is not able to be phosphorylated and thereby inhibits the activation of the NF-κB pathway, similar findings have been demonstrated in that suppressive NF-κB radiosensitizes prostate cancer cells (6). However, the role of the NF-κB pathway in regulating the radiosensitivity remains controversial in different diseases. Jung *et al* demonstrated that by transfecting a phosphorylation-defective IκBα gene into cells derived from a patient with ataxia telangiectasia (AT) group D, the constitutive activation of NF-κB was suppressed and the previous radiation hypersensitivity in AT was restored to the normal level (11). Additionally, to the best of our knowledge, the impact of this mutant IκBα gene on the expression of NF-κB and subsequent changes in radiosensitivity in ACC cells had not yet been studied.

In the present study, we investigated the role of NF-κB in regulating the radiosensitivity of adenoid cystic carcinoma cells (ACC-M) *in vitro*. To achieve a conclusion, we transiently transfected a plasmid encoding a Flag-tagged phosphorylation defective mutant of IκBα (S32, 36A) (pBαbe-SR-IκBα) into ACC-M cells, thereby suppressing the activity of the NF-κB pathway. While analyzing the results, we observed that suppressing the NF-κB pathway by transfecting ACC-M cells with mutant IκBα, as compared with cells with a control pBαbe plasmid transfection, leads to increased radiosensitivity. Meanwhile, intragroup data analysis of the SR-IκBα group demonstrated that different doses of irradiation induced the expression of NF-κB in a dose- and time-dependent manner, with corresponding changes in the radiosensitivity of ACC-M cells.

## Materials and methods

### Chemicals and reagents

The pBαbe-SR-IκBα and control pBαbe plasmids were provided by Professor Wantao Chen (Department of Oral and Maxillofacial Surgery, Ninth People’s Hospital, College of Stomatology, Shanghai Jiao Tong University, Shanghai, China). Lipofectamine was purchased from Invitrogen Life Technologies (Carlsbad, CA, USA).

An MTT and an Annexin V-FLUOS staining kit were purchased from Roche Diagnostics (Indianapolis, IN, USA). Rabbit anti-human NF-κB, goat anti-rabbit secondary antibodies, anti-β-actin and horseradish peroxidase (HRP)-conjugated goat anti-rabbit immunoglobulin were purchased from Santa Cruz Biotechnology, Inc. (Santa Cruz, CA, USA). Anti-IκBα antibody was purchased from Cell Signaling Technology, Inc. (Denvers, MA, USA).

### Cell culture

The human salivary adenoid cystic carcinoma cell line (ACC-M) was provided by Professor Wantao Chen. ACC-M cells were cultured in RPMI-1640 medium supplemented with 10% filtered fetal bovine serum (FBS; Hyclone, Logan, UT, USA) and 100 U/ml penicillin/streptomycin. The cultures were incubated at 37°C in a humidified 5% CO_2_ incubator.

### Plasmid transfection

The site-specific, signal-induced degradation of IκBα depends on phosphorylation at Ser 32 and 36. Therefore, the pBαbe-SR-IκBα plasmid that consisted of a double point mutation (Ser to Lactamine) was thus resistant to phosphorylation (12). The mutant and control plasmids were transiently transfected into ACC-M cells with the use of Lipofectamine, according to the manufacturer’s instructions. In brief, ACC-M cells were removed by trypsin/EDTA treatment and seeded at a density of 2×10^5^ cells/ml in 6-cm culture dishes. Cells were grown to 90% confluence and subjected to 24-h synchronization in serum-free medium. ACC-M cells were transfected with 4 *μ*g of the pBαbe-SR-IκBα or control pBαbe plasmid per dish with the use of Lipofectamine. Following incubation for 6 h, the transfection medium was replaced by fresh medium for an additional 48-h incubation to allow for gene expression to occur.

### MTT

The effect of transfection on cellular proliferation was assessed using MTT. In brief, the non-transfected, transfected pBαbe-SR-IκBα or control plasmid cells were seeded in 96-well plates in fresh medium (5000 cells/well) and incubation was continued for an additional 24, 48 or 72 h following transfection. Thereafter, MTT solution (5 mg/ml) was added to each well. Following incubation for 4 h at 37°C, the blue dye taken up by the cells was dissolved in dimethyl sulfodide (100 *μ*g/ml), and then the optical density was measured at 570 nm using a 96-well multiscanner. All assays were run in triplicate.

Cells in the different groups mentioned previously were irradiated at room temperature with a medical linear accelerator (Primus-H; Siemens, Munich, Germany) at different doses. Cells in the transfected pBαbe-SR-IκBα plasmid group were harvested at different time points (1, 3, 6, 10, 24 and 48 h) following exposure to graded irradiation doses (0, 2, 4, 6, 8 and 10 Gy). Cells in the pBαbe and non-transfected groups were harvested 3 h following exposure to graded irradiation doses. The 3 h time point was selected due to the fact that, as described by Sandur *et al*, the maximum expression of NF-κB occurrs 3 h following irradiation (10). Treated cells in the indicated groups were prepared for sequential immunocytochemistry, as well as western blot and flow cytometric analyses.

### Immunocytochemistry

Changes in NF-κB nuclear translocation were examined by immunocytochemistry. In brief, cells were plated onto 10×10-mm glass slides, and subjected to transfection and irradiation treatment. Thereafter, cells were fixed with 4% paraformaldehyde and permeabilized with 0.2% Triton X-100. Following washing in phosphate-buffered saline (PBS), slides were blocked with 5% normal goat serum for 1 h and then incubated with rabbit anti-NF-κB antibody at a dilution of 1:100. Following overnight incubation at 4°C and rewarming to 37°C, the slides were washed with PBS and incubated for 20 min with goat anti-rabbit secondary antibody at a dilution of 1:100. The slides were then washed in PBS and visualized using diaminobenzidine. Negative controls for each group were processed in the same manner, using a non-immunized rabbit IgG (at a dilution of 1:100) in place of the primary antibody. Immunocytochemical staining for NF-κB in the nucleus was quantitatively analyzed using the Image-Pro Plus image analytical system (Media Cybernetics; Silver Spring, MD, USA) with the gray scale method.

### Western blot

The changes in the expression of NF-κB and mutant IκBα following transfection and irradiation treatment were evaluated by western blot analysis. In brief, whole cell extracts were prepared in RIPA buffer (50 mM Tris-HCl, pH 7.4; 150 mM NaCl; 0.5 sodium deoxycholate; 0.1% SDS; 1% NP40 and 1 mM phenylmethylsulfonyl fluoride; Shenneng Bocai Biotechnology Co., Ltd.; Shanghai, China) from the treated cells grown in 6-cm dishes. The protein concentration was quantified using the BCA protein measurement kit (Shenneng Bocai Biotechnology Co., Ltd.). Extracts (40 *μ*g) were subjected to SDS-PAGE and then electrophoretically transferred to nitrocellulose membranes, which were then blocked with 5% (w/v) dried skimmed milk-TBST (10 mm Tris-HCl, pH 8.0; 150 mm NaCl; 0.05% Tween-20) for 1 h at room temperature. Membranes were probed with anti-NF-κB antibody (at a dilution of 1:1000) or anti-IκBα antibody (at a dilution of 1:1000), incubated overnight in 5% milk-TBST, washed three times with 1X TBST and exposed to HRP-conjugated secondary antibody (at 1:10000 dilution) in 5% dried skimmed milk for 1 h. All blots were reprobed with anti-β-actin (at 1:1000 dilution) in 5% dried skimmed milk, followed by HRP-conjugated secondary antibody (at a dilution of 1:1000) in 5% dried skimmed milk. Protein bands were visualized by the Alpha Imager 2200 system (Alpha Innotech, San Leandro, CA, USA).

### Flow cytometric analysis

The effects on apoptosis in cells following the treatment indicated previously were determined by flow cytometric analysis. In brief, cells were trypsinized, then harvested by centrifugation at 1000 × g for 3 min. Thereafter, the cell collections were washed twice with cold PBS, resuspended in Annexin V and propidium iodide (PI), and incubated for 15 min at 4°C. Fluorescence was measured at 488 nm in a flow cytometer (Coulter, Luton, UK). Early apoptotic cells are indicated by Annexin V-positive and PI-negative staining, whereas late apoptotic cells demonstrate both Annexin V- and PI-positive staining.

### Statistical analysis

SPSS/PC (version 17.0; SPSS, Inc., Chicago, IL,USA) was used for statistical analysis. Data are expressed as means ± standard deviation and were analyzed by a one-way ANOVA with an LSD test. The Pearson’s correlation coefficient analysis was used to assess the correlation between radiation doses, NF-κB expression and apoptotic rate. P<0.05 was considered to indicate a statistically significant difference.

## Results

### Expression of IκBα protein is affected following transfection in ACC-M cells

**Figure 1:**
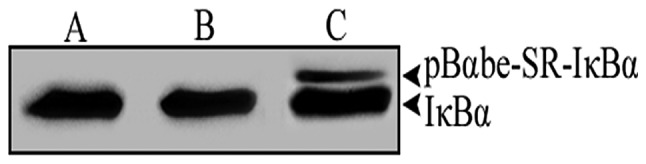
Expression of IκBa protein following pBabe-SR-IκBa plasmid transfection, demonstrated by western blot analysis; (A) the non-transfected, (B) pBαbe and (C) pBαbe-SR-IκBα groups.

To examine the changes in NF-κB following irradiation in ACC-M cells, we transiently transfected the cells by use of a pBαbe-SR-IκBα plasmid prior to irradiation, and thus suppressed the activity of the NF-κB pathway. The expression of the Flag-tagged IκBα following transfection was confirmed by western blot analysis. As demonstrated in Fig. 1, in the pBαbe and non-transfected groups, cells exhibited a major band corresponding to endogenous IκBα protein. However, the ACC-M cells that had been transfected with the pBαbe-SR-IκBα plasmid expressed endogenous IκBα protein, as well as the mutant IκBα protein with a higher molecular weight.

### In vitro growth properties of transfected ACC-M cells

**Figure 2:**
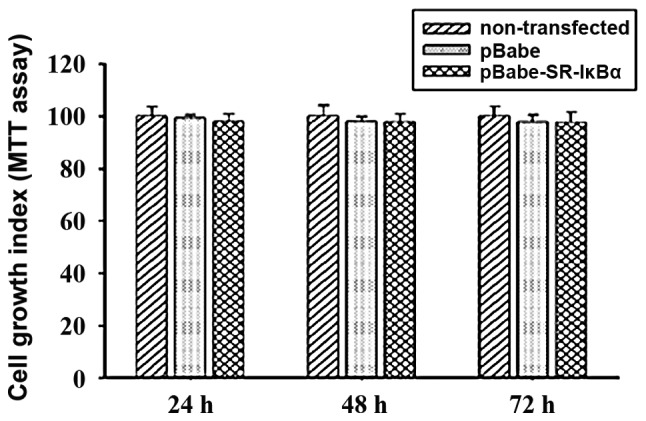
Effects of pBαbe-SR-IκBα gene transfection on the growth of adenoid cystic carcinoma cell line (ACC-M) cells were measured by MTT assay.

To determine whether the mutant IκBα gene exerted its antiproliferative activity on the transfected cells, cells were subjected to an MTT analysis. As demonstrated in Fig. 2, no visible transfection-related inhibition was observed between the pBαbe and non-transfected groups, while the growth property of cells in the pBαbe-SR-IκBα group was slightly reduced to 98.07, 97.79 and 97.5% after 24, 48 and 72 h, respectively. However, no significant difference was observed between the pBαbe-SR-IκBα group and the pBαbe or non-transfected groups at the 95% probability level. Accordingly, it is likely that the expression of mutant IκBα protein does not necessarily lead to the *in vitro* growth inhibition of non-stimulated ACC-M cells.

### Translocation of NF-κB following irradiation in ACC-M cells

NF-κB staining was located within the cell cytoplasm in resting cells. Following exposure to different doses of irradiation, the previously suppressed NF-κB pathway (achieved by transfecting the ACC-M cells with mutant IκBα) was activated and thereafter liberated NF-κB translocated to the nucleus. The staining of NF-κB in the nucleus was categorized as positive expression. Compared with the pBαbe and nontransfected groups at 3 h following exposure to equal doses of irradiation, the NF-κB pathway was notably inhibited in the pBαbe-SR-IκBα group, as demonstrated by a relatively lightly stained nucleus and the fact that the immunoprecipitates were mainly distrubuted in the cytoplasm. The majority of the NF-κB protein in the cells of the pBαbe-SR-IκBα group was found to be clustered in the cytoplasm around the nuclear envelope. With exposure to graded doses of irradiation, the NF-κB protein was observed to pass through the nuclear envelope and gradually accumulate in the nucleus. The NF-κB protein was first observed in the center of the nucleus at 6 h (data not shown).

**Figure 3:**
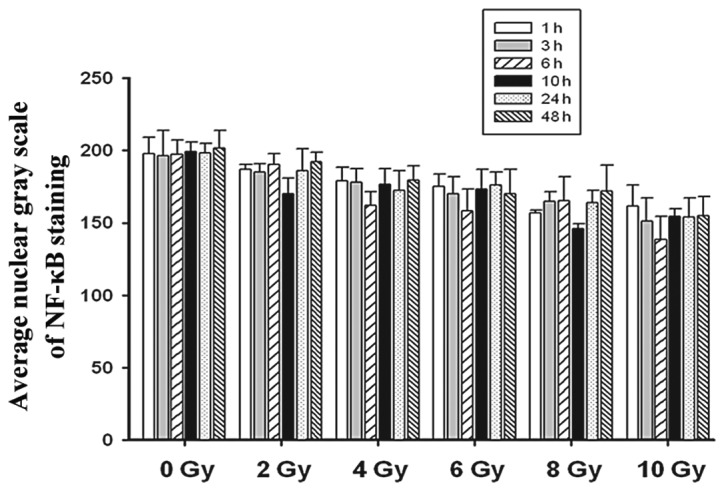
Average nuclear gray scale at different time points following exposure to graded doses of irradiation in the pBαbe-SR-IκBα group.

The quantitative analysis of the immunocytochemistry images supported our findings. Compared with the pBαbe and non-transfected groups at 3 h following exposure to equal doses of irradiation, the average nuclear gray scale of cells in the pBαbe-SR-IκBα plasmid group was significantly increased (P<0.05; data not shown). As demonstrated in Fig. 3, further analysis of the SR-IκBα group revealed that the average nuclear gray scale of the various dose groups was lower than that of the 0 Gy group with only marginally detectable expression of NF-κB protein, and the data reached the deepest valley 6–10 h following irradiation. At the same time point, we disclosed that at 3 h following irradiation, the average nuclear gray scale of the ACC-M cells decreased as the doses of irradiation increased (P<0.05).

### Expression of NF-κB protein is affected following irradiation in ACC-M cells

**Figure 4:**
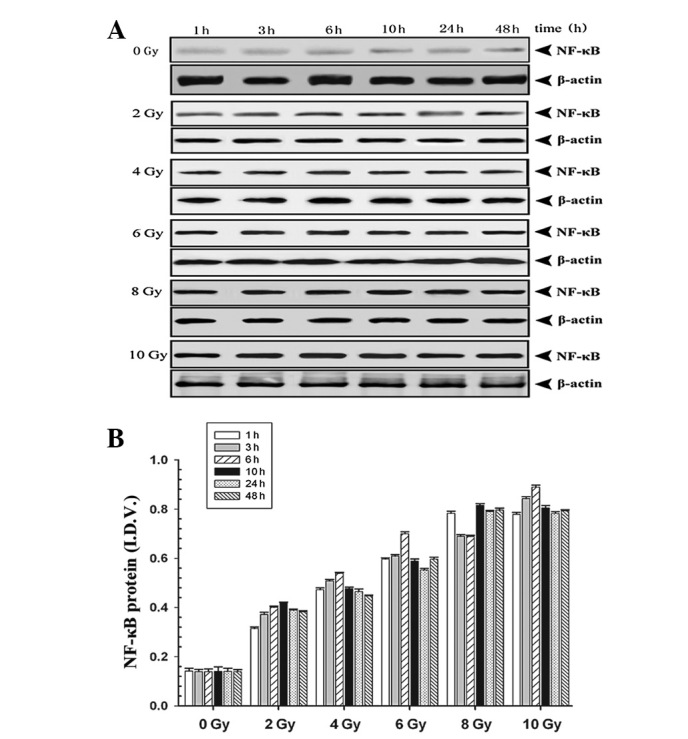
(A) Quantitive changes in NF-κB expression at different time points following exposure to graded doses of irradiation, as demonstrated by western blot analysis in the pBαbe-SR-IκBα group. (B) The relative level of NF-κB expression in 40 *μ*g of whole cell extract as determined by western blot analysis. Data are presented as the mean integrated density value (IDV) of three separate experiments (± standard deviation).

To explore the quantitative changes in NF-κB protein levels in ACC-M cells, cell lysates were subjected to western blot analysis following transfection and subsequent irradiation. Cells in the pBαbe-SR-IκBα plasmid group demonstrated downregulated levels of NF-κB protein (P<0.05), which suggested transfection of mutant IκBα simultaneously blocked the synthesis and translocation of NF-κB protein (data not shown). Further analysis on the time course and dose-response characteristics of NF-κB protein in the pBαbe-SR-IκBα plasmid group are demonstrated in Fig. 4. The levels of NF-κB protein in the various dosage groups were significantly higher than that of the 0 Gy group. The NF-κB protein levels peaked at 6–10 h and began gradually declining at 24 h in each group with equal irradiation exposure. At the 3 h time point, the levels of NF-κB protein increased as the dose of irradiation increased (P<0.05). Overall, the western blot analysis results are consistent with our immunocytochemistry data.

### Changes in the apoptotic rate following irradiation in ACC-M cells

**Figure 5:**
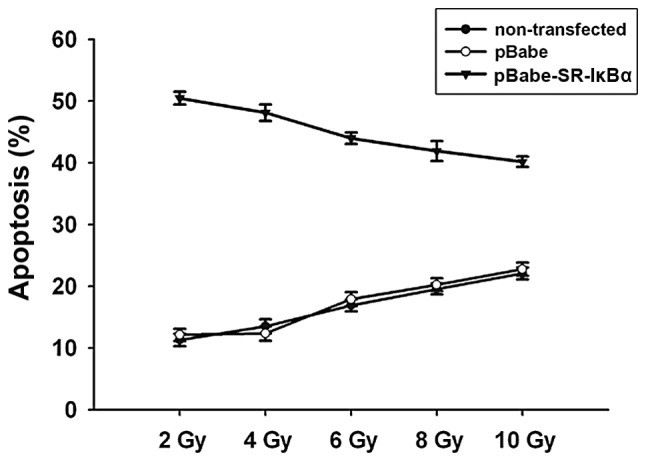
Rate of apoptosis in the different transfection groups, as measured by flow cytometery.

**Figure 6:**
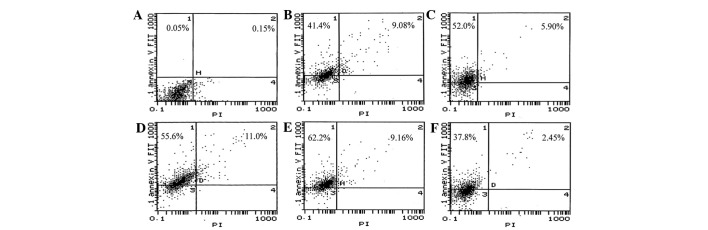
Apoptosis of adenoid cystic carcinoma cell line (ACC-M) cells following irradiation in the pBαbe-SR-IκBα group, which comprised the following groups: (A) Control; (B) 2 Gy, 3 h; (C) 4 Gy, 24 h; (D) 6 Gy, 24 h; (E) 8 Gy, 24 h; and (F) 10 Gy, 3 h.

**Figure 7:**
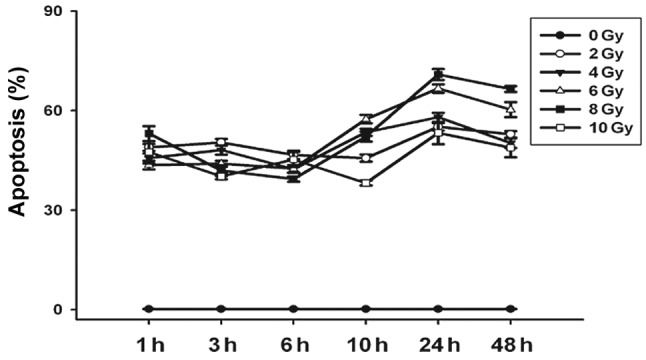
Apoptosis of adenoid cystic carcinoma cell line (ACC-M) cells following exposure to graded doses of irradiation in the pBαbe-SR-IκBα group at different time points, demonstrated by flow cytometery.

It has been demonstrated that irradiation results in deleterious DNA damages and consequentially launches cell apoptosis. However, the simultaneously triggered cellular defense mechanism prevents impaired cells from the radiation-induced apoptosis (13,14). NF-κB has been demonstrated to be activated by irradiation, and to therefore elucidate the elevated irradiation resistance (6). As demonstrated in Fig. 5, treatment with mutant IκBα transfection significantly enhanced the radiosensitivity of ACC-M cells, as assessed by flow cytometric analysis (P<0.05). A significant increase in the number of both Annexin V-positive and PI-negative (early apoptosis) and Annexin V- and PI-positive (late apoptosis) ACC-M cells was detected in the pBαbe-SR-IκBα plasmid group with graded doses of irradiation, compared with the 0 Gy group (Fig. 6). Furthermore, cells with equal irradiation exposure exhibited a similar survival tendency in that the apoptotic rate reached the minimal value at 6–10 h, and the maximal value at 24 h. The apoptotic rates of cells with mutant IκBα transfection, 3 h following irradiation treatment, were 50.83, 48.61, 44.32, 42.53 and 40.42%, between the different dosage groups (2, 4, 6, 8 and 10 Gy, respectively). These results suggested that the increasing expression of NF-κB was attributed to the decreasing number of total (early and late) apoptotic cells at 3 h (Fig. 7).

The linear correlation estimation was applied to demonstrate that the apoptotic rates correlated well with the quantitative changes in NF-κB protein expression. The apoptotic rate of the cells was significantly negatively correlated with the expression of NF-κB protein. The overexpression of NF-κB protein 6–10 h following irradiation in the pBαbe-SR-IκBα plasmid group led to the lowest rate of apoptosis. Meanwhile, graded doses of irradiation amplified the activation of the NF-κB pathway, which in reverse decreased the apoptotic rates at 3 h, and the increased expression of NF-κB at 3 h decreased the apoptotic rates.

## Discussion

The primary aim of our study was to determine whether the transfected mutant IκBα gene was able to inhibit the activity of the NF-κB pathway, and to investigate the negative contribution of the radio-activated NF-κB pathway on radio-sensitivity. We transiently transfected a mutant IκBα gene into ACC-M cells, obtaining simultaneous blockage of the synthesis and translocation of the NF-κB protein. The mutant IκBα gene promoted radiosensitivity via suppression of the NF-κB pathway. Additionally, exposure to radiation invoked the activity of the NF-κB pathway that was initially inhibited by transfection, in a dose- and time-dependent manner. The highly active NF-κB protein in reverse exhibited a radio-resistant effect in the ACC-M cells and improved survival following irradiation.

NF-κB plays a pivotal role in various physiological processes, including immune and inflammatory responses, and is also considered to regulate apoptosis, cell proliferation and resistance to chemotherapy- and radiotherapy-induced cytotoxicity (15). NF-κB is sequestered inactive in the cytoplasm by interaction with an inhibitor of IκBα in resting cells. It is now widely accepted that the degradation of IκBα plays a crucial role in the activation of the NF-κB pathway (12). When cells were stimulated by different exogenous stimuli, the Ser 32 and 36 residues of IκBα were phosphorylated by IKK and IκBα underwent subsequent ubiquitylation and degradation, thus releasing the NF-κB protein for nuclear translocation (16). Fujioka *et al* transfected a retroviral vector encoding phosphorylation-defective IκBα by inducing point mutations at the potential phosphoacceptor sites of Ser 32 and 36, and observed decreased NF-κB DNA binding activity in the non-metastatic human pancreatic tumor cell line (17). However, to the best of our knowledge, applying a mutant form of IκBα to specifically inhibit the NF-κB pathway in ACC-M cells has not been previously studied. As we have demonstrated in ACC-M cells, the synthesis and translocation of the NF-κB protein were highly specifically suppressed by transfecting the cells with the use of a pBαbe-SR-IκBα plasmid, and this blocking effect persisted following irradiation and hence when the NF-κB pathway was activated.

Given the involvement of the NF-κB pathway in oncogenesis and induction of resistance to conventional therapy, such as chemotherapy and irradiation, blockage of the pathway by specific inhibitors is beneficial and may be a promising antitumor therapy (18). NF-κB is suppressed by multiple strategies, including the application of a phosphorylation-defective form of IκBα, proteasome inhibitors or pharmacological agents (10,17,18). van Hogerlinden *et al* applied a super-repressor form of IκBα with mutant phosphorylation sites that selectively inhibited the NF-κB pathway, yielding elevated apoptosis in the basal and suprabasal keratinocytes *in vivo*(19). In addition, the super-repressor considerably sensitized the keratinocytes to UV-induced apoptosis. Duffey *et al* also demonstrated that stable expression of a mutant form of IκBα in squamous carcinoma cells resulted in an augmented apoptotic rate, and this blocking effect was not relieved by TNF-α induced activation (20). However, in the present study, no significant effects on cell proliferation were observed in ACC-M cells with pBαbe-SR-IκBα plasmid transfection. By contrast, cells with prior mutant IκBα plasmid transfection were susceptible to radio-induced apoptosis. Accordingly, we estimate that inhibition of the NF-κB pathway in non-stimulated ACC-M cells does not necessarily result in increased apoptosis, whereas inhibition of the NF-κB pathway combined with irradiation yielded a significant radiosensitizing outcome.

Cytoplasmic NF-κB is activated by various stimuli, including IL-1, TNF-α and viral infection, as well as irradiation and chemotherapeutic agents (8). Li *et al* demonstrated that maslinic acid, a triterpene derivative obtained from olive pomace, inhibited endogenous NF-κB activity in a time-dependent manner and the TNFα-induced NF-κB activation in a dose-dependent manner in pancreatic cancer cells, and thus prevented the expression of downstream target genes, such as Bcl-2, Survivin, COX-2 and IAP-1 (21). Sandur *et al* also demonstrated that maximal expression of NF-κB occurred 3 h following exposure to 10 Gy irradiation in colorectal cancer cells (10). However, there are a limited number of studies concerning the time-dose expression pattern of radio-induced activation in previously inhibited NF-κB pathway. In our study, a similar expression tendency was observed in the pBαbe-SR-IκBα plasmid group; the maximal expression appeared at 6–10 h following irradiation. At 3 h, graded doses of irradiation corresponded well with the increased activity of the NF-κB pathway.

Accumulating studies indicate that the constitutive activation of NF-κB is closely correlated with oncogensis and tumor invasion. Aberrant activation of the NF-κB pathway has been identified in various types of cancer, including both solid and hematopoietic malignancies (22). Zhang *et al* revealed that an activated NF-κB pathway was significantly correlated with advanced clinical stage, high frequencies of tumor angiogenesis and perineural invasion, aggressive locoregional recurrence and distant metastasis in ACC, all of which predict an adverse prognostic prospect (23). Sustained activation of the NF-κB pathway has been demonstrated to promote survival in tumor cells with radiotherapy by permitting cells to repair DNA damage and to escape elimination by apoptosis, which is induced by the destruction of DNA double-strand (24). In our study, the peak expression levels of NF-κB protein at 6–10 h following irradiation were paralleled by minimal apoptotic rates in the pBαbe-SR-IκBα plasmid group. Amplified expression levels of NF-κB activated by graded doses of irradiation at 3 h reflected attenuated apoptosis. These findings to an extent explain the radio-resistant role of NF-κB in stimulated ACC-M cells.

In the present study, we demonstrated that ACC-M cells exhibited markedly increased radiosensitivity following selective inhibition of the NF-κB pathway by transfection with a mutant IκBα plasmid. However, the NF-κB pathway also plays a crucial role in various physiological processes, such as regulation of innate immunity by activating downstream pro-inflammatory cytokines, including IL-1, TNF-α and RANTES. One of the pitfalls that may be neglected is the likelihood of immunodeficiency and great susceptibility to infections following specific inhibition of the NF-κB pathway. The correlation between downregulation of the NF-κB pathway and human diseases is reported in *Incontinentia pigmenti* and anhidrotic ectodermal dysplasia (25). van Hogerlinden *et al* observed interferences in normal epidermal homeostasis and hair follicle development in selectively inhibited genetic mice (19). The transgenic mouse model exhibited dysplasia of the epidermis characterized by flaky skin, hair loss and hyperkeratotic lesions. The increased susceptibility to the spontaneous occurrence of well-differentiated skin squamous cell carcinoma was also demonstrated in the transgenic mice. Accordingly, the appropriate application of NF-κB pathway inhibitors while avoiding their side-effects requires further investigation.

In conclusion, the results of our study demonstrated that by transfecting a mutant form of the IκBα gene, the synthesis and translocation of the NF-κB protein were specifically inhibited, and a concomitant decrease in survival rate following irradiation was observed in ACC-M cells. Furthermore, the NF-κB expression after irradiation represents a time-dose dependent manner of the transfected cells, followed by a corresponding change in radiosensitivity. Accordingly, selective inhibition of the NF-κB pathway by novel target agents reveals a promising method for improvements in the radiosensitivity of ACC. However, the potential side-effects that may be activated by systemic suppression of immune system require further investigation.
